# The Anti-atherosclerotic Effect of Paeonol against Vascular Smooth Muscle Cell Proliferation by Up-regulation of Autophagy via the AMPK/mTOR Signaling Pathway

**DOI:** 10.3389/fphar.2017.00948

**Published:** 2018-01-04

**Authors:** Hongfei Wu, Aiwei Song, Wenjun Hu, Min Dai

**Affiliations:** ^1^School of Pharmacy, Anhui University of Chinese Medicine, Hefei, China; ^2^Key Laboratory of Xin’an Medicine, Ministry of Education, Hefei, China

**Keywords:** Paeonol, atherosclerosis, cell proliferation, autophagy, AMPK/mTOR pathway

## Abstract

**Introduction:** Paeonol (2′-hydroxy-4′-methoxyacetophenone), isolated from moutan cortex, is an active component and has been shown to have anti-atherosclerotic and anti-proliferation effects on vascular smooth muscle cells (VSMCs). However, the possible role of Paeonol in protecting against VSMC proliferation as related to autophagy has yet to be elucidated.

**Materials and Methods:** The athero-protective effects of Paeonol were evaluated in apoE^-/-^ mice. The effects of Paeonol on VSMC proliferation and autophagy were examined by staining α-SMA and LC3II spots in the media layer of apoE^-/-^ mice, respectively. CCK8 and BrdU assays were used to investigate the effects of Paeonol on cell proliferation *in vitro*. The autophagic levels in VSMCs were evaluated by detecting LC3II accumulation and p62 degradation by immunoblot analysis. To investigate if Paeonol could prevent VSMCs proliferation through autophagy induction, we tested the change in autophagy and cell proliferation by inhibition of autophagy. The levels of the AMPK/mTOR pathway in autophagy regulation were detected by immunoblot analysis. An AMPK inhibitor and si-AMPK transfection in VSMCs was used to confirm whether AMPK activity plays a key role in autophagy regulation of Paeonol.

**Results:** *In vivo* experiments confirmed that Paeonol restricted atherosclerosis development and decreased the amount of VSMCs in the media layer of apoE^-/-^ mice. Paeonol increased protein levels of LC3II and the presence of autophagosomes in the media layer of arteries, which implies that Paeonol may induce VSMCs autophagy *in vivo*. Paeonol showed potential in inhibiting ox-LDL-induced proliferation *in vitro* experiments. Paeonol dose-dependently enhanced the formation of acidic vesicular organelles and autophagosmomes, up-regulated the expression of LC3II and increased p62 degradation. The autophagy inhibitor CQ obviously attenuated Paeonol-induced autophagy and the anti-proliferation effect in VSMCs. In addition, Paeonol induced phosphorylation of AMPK and reduced phosphorylation of mTOR. An AMPK inhibitor reversed the Paeonol-induced p-mTOR/mTOR decrease. Paeonol induced LC3II conversion, increased p62 degradation and inhibited cell proliferation in VSMCs, the effects of which were abolished by si-AMPK.

**Conclusion:** These results imply that Paeonol inhibits proliferation of VSMCs by up-regulating autophagy, and activating the AMPK/mTOR signaling pathway, providing new insights into the anti-atherosclerosis activity of Paeonol.

## Introduction

Atherosclerosis is a complex chronic vascular disease that is typically characterized by atherosclerotic plaques in the arterial walls and can narrow blood vessels (Lusis, 2000; Libby, 2002; Baldrighi et al., 2017). Excessive vascular smooth muscle cell (VSMC) proliferation has proven to be very important during atherogenesis in animal models and in human studies (Fuster et al., 2010; Lacolley et al., 2012). VSMC proliferation leads to the growth of plaques in the intima and the media layer of arteries, resulting in partial or total obstruction of the vascular lumen (Hansson, 2005). Surgical anti-proliferation treatments, including angiogenesis, implanting stents and performing bypass, have been employed to restore the occluded vascular lumen. However, these strategies have often failed because of VSMC-induced in-stent restenosis, a serious complication in treatment (Mills et al., 2012). Increased cell proliferation within the arterial wall is a crucial contributor to plaque formation and restenosis. Therefore, understanding the regulatory mechanisms that control proliferation of VSMCs is important for efficient therapies against atherosclerosis.

Autophagy is a conserved cellular process associated with the degradation of long-lived proteins and dysfunctional organelles (Yoshimori, 2004; Yin et al., 2016). Autophagy inhibits VSMC proliferation *in vitro*, which is caused by multifarious atherogenic stimuli (Dong et al., 2012; Hu et al., 2012). The majority of drug-eluting stents are commonly loaded with rapamycin-based drugs to induce autophagy, which decreases VSMC proliferation and promotes a contractile phenotype (Grube and Buellesfeld, 2004). Likewise, verapamil and emodin activate autophagy and induced anti-proliferation effects in VSMCs (Salabei et al., 2012; Salabei and Conklin, 2013). Recent studies indicated that VSMC autophagy was of vital importance during the process of atherogenesis (Tai et al., 2016; Bravo-San Pedro et al., 2017). During the initial atherogenesis period, up-regulation of autophagy in VSMCs was conducive to prompting a quiescent cellular phenotype, reducing proliferation and inhibiting fibrosis (Wei et al., 2013). In addition, defective autophagy in VSMCs disturbed cell homeostasis and induced cell proliferation, which finally led to and even accelerated atherogenesis (Grootaert et al., 2015). Although few studies to date have examined the direct role of VSMCs autophagy in pathological processes, autophagy might be an adaptive strategy to prevent cell proliferation and treat atherosclerosis.

Paeonol (2′-hydroxy-4′-methoxyacetophenone), isolated from moutan cortex, is an active component and has been shown to have anti-tumor, anti-inflammation, anti-atherosclerotic, and anti-proliferation effects on VSMCs (Sun et al., 2008; Pan and Dai, 2009; Chen et al., 2014; Zhang et al., 2015). Despite these pharmacological findings, the possible role of Paeonol in protecting against VSMC proliferation as related to autophagy has yet to be elucidated. In the present study, we attempted to explore the role of autophagy in VSMC proliferation and clarify the underlying mechanisms of a protective autophagic pathway *in vivo* and *in vitro*.

## Materials and Methods

### Ethics Statement

All surgical and experimental procedures were approved by the Ethics Review Committee for Animal Experimentation of the Institute of Clinical Pharmacology at Anhui Medical University.

### Chemicals and Reagents

Paeonol (98.5%) was purchased from Baicaoplants Biotechnology Company (Anhui, China). Ox-LDL was purchased from Xinyuanjiahe Biotechnology Company (Bejing, China). CCK-8 and BrdU cell proliferation kits were purchased from the Beyotime Institute of Biotechnology (Shanghai, China) and Roche Diagnostics GmbH (Mannheim, Germany), respectively. The following antibodies were used in this study: PCNA (ab92552, Abcam), LC3 (ab128025, Abcam), p62 (ab56416, Abcam), AMPK (ab80039, Abcam), mTOR (ab32028, Abcam), p-AMPK (ab133448, Abcam), p-mTOR (5536, Cell signaling), and α-SMA (ab7817, Abcam). Horseradish peroxidase-conjugated secondary antibodies were purchased from Zhongshan Jinqiao Biotechnology Co, Ltd. (Bejing, China). The secondary antibodies for immunofluorescence were goat anti-mouse IgG Alexa Fluor-488 and goat anti- rabbit IgG Alexa Fluor-594 (Invitrogen, Carlsbad, CA, United States). All other chemical reagents were obtained from commercial vendors.

### Animal Model

ApoE-knockout mice on a C57BL/6 background were purchased from the Department of Laboratory Animal Science, Vital Riverv, Beijing, China, and used to build the atherosclerosis animal model. Male mice aged 6 weeks were kept under standard laboratory conditions (temperature 22–25°C and relative humidity 55–65%) with 12:12 dark/light. The mice were fed a high-cholesterol diet (HCD, containing 21% fat and 0.15% cholesterol) until atherosclerotic lesions obviously formed on arteries of mice. Subsequently, atherosclerotic animals were randomly put into four groups (*n* = 8 mice/group). Three group were orally administered with 400, 200, and 100 mg/kg body weight of Paeonol in a 5% CMC-Na solution daily for a total of 6 weeks. One group of mice was orally administered with CMC-Na solution as the model group, and 6-week-old male C57BL/6 mice were fed a normal diet throughout the experiment as the control group.

Mice were sacrificed by exsanguination. Blood samples were collected, and hearts as well as arteries were removed and placed into cold PBS. The arteries were cleaned from the surrounding connective tissue and embedded in OCT embedding medium for histology and immunofluorescence assays.

### Serum Biochemical Assays

The concentration of total cholesterol (TC) and triglycerides (TG) was measured by using commercial kits (DIASYS company, Shanghai, China). LDL-cholesterol (LDL-c) and HDL-cholesterol (HDL-c) were analyzed by enzymatic colorimetric methods using commercial Kits (SEKISUI company, Shanghai, Japan). The level of ox-LDL was measured with an ELISA kit (Biosource, United States). These assays were performed in a blinded manner and in duplicates within each animal group.

### Histology and Immunofluorescence

Arterial sinuses were embedded in OCT embedding medium, cut at 7 μm thickness and stained with haematoxylin and eosin (H&E). The arterial root, 10 μm, underwent Oil-red O (ORO) staining. Frozen sections were processed and incubated with anti-α-SMA antibody (1:200 dilution) and anti-LC3 antibody (1:200 dilution). LC3 and α-smooth muscle actin (α-SMA) were visualized with goat anti-mouse IgG Alexa Fluor-488 (1:500 dilution) and goat anti- rabbit IgG Alexa Fluor-594 (1:500 dilution), respectively. The images were captured by a confocal microscope (SP5, Leica, Germany).

### En Face Staining of Aorta

Whole arteries, including aortic arch, thoracic, and abdominal regions, were cut longitudinally, fixed and then stained with ORO for lipid measurement at the surface of the vascular wall. The images were captured by a digital camera (Canon EOS 7D, Tokyo, Japan).

### Cell Culture and Treatment

Vascular smooth muscle cells were isolated from C57BL/6J mouse arteries by an explant technique as our previous research described (Liu et al., 2014). In short, arteries were opened longitudinally, and the endothelium and vascular adventitia were slightly scraped. The media layer of the arteries was digested in collagenase I solution, cut into pieces and then placed into culture flasks. The pieces of arteries were incubated in DMEM containing fetal calf serum at 37°C in 5% CO_2_. After cell confluence, the cells were subcultured. Immuno-histochemistry for α-SMA (positive) and Factor VIII (negative) was used to confirm VSMC purity. All experiments involved VSMCs at no more than passage 10. For the inhibition test, VSMCs were treated with chloroquine (CQ, 50 μM), compound C (CC, 10 μM) and rapamycin (rap, 100 nM) for 1h following the addition of ox-LDL incubation with or without Paeonol for 24 h.

### Assessment of Cell Viability via CCK-8 Proliferation Assay

The effect of different concentrations (12.5–200 μg/mL) of ox-LDL on VSMC proliferation was assessed by incorporation of CCK-8. Briefly, VSMCs were seeded into 96-well plates and incubated with different concentrations of ox-LDL for 24 h. CCK-8 solution was added and incubated in the dark for 1.5 h, and then they were tested by a microplate absorbance reader at 450 nm.

### Assessment of Cell Proliferation via BrdU Assay

Vascular smooth muscle cells were treated with ox-LDL (50 μg/mL) in the presence or absence of Paeonol at various concentrations (7.5, 15, 30, 60, 120, and 240 μM) for 24 h. According to the manufacturer’s instructions, BrdU was added 1 h before the end of the experiment. The relative proliferation ratio was absorbance of the treated VSMCs to the absorbance of control VSMCs. BrdU incorporation of the control group was set to 100%.

### Cell Cycle Analysis via Flow Cytometry

The treated VSMCs were collected and incubated with 70% methanol at -20°C for 24 h. Cells were re-suspended with propidium iodide (PI) solution containing Triton X-100, sodium citrate and PI (50 μg/mL). PI-stained VSMCs were examined by flow cytometry (BD Accuri C6, Becton-Dickinson, Mansfield, MA, United States). Cell percentages were analyzed using BD Accuri C6 softwares (Version 1.0.264, Mansfield, MA, United States).

### Cell Staining for Immunofluorescence Microscopy

Vascular smooth muscle cells were incubated with anti-LC3 antibody and anti-p62 antibody (both at 1:500), followed by incubation with the corresponding secondary antibodies. Cells were then incubated with DAPI to visualize the nucleus. Finally, cells were evaluated by inverted fluorescence microscopy.

### Cell Staining with Acridine Orange (AO)

Vascular smooth muscle cells were treated with AO (1 μg/mL) for 15 min at 37°C in the dark. VSMCs were observed under an inverted fluorescence microscope. Alternatively, AO-stained VSMCs were collected and analyzed by flow cytometry. Accumulation of acidic vesicular organelles was quantified as the red/green fluorescence ratio (FL3/FL1 mean).

### Transmission Electron Microscopy (TEM) Analysis

Samples were fixed in 2.5% glutaraldehyde and post-fixed by 2% OsO_4_. After dehydration in graded alcohols, samples were embedded in Epon-Araldite resin. Thin sections were stained with uranyl acetate and lead citrate. A transmission electron microscope (transmission electron microscopy, TEM, H-7650, Hitachi, Japan) was utilized to observe autophagosomes.

### Si-RNA Technique

Small interfering RNAs against mouse AMPK-α1 (si-AMPK sequences, sense: 5′-GAG CGA CUA UCA AAG ACA UTT-3′, anti-sense: 5′-AUG UCU UUG AUA GUC GCU CTT-3′) and scrambled si-RNA were used according to manufacturer’s protocol and were purchased from Santa Cruz Biotechnology.

### Western Blot

Vascular smooth muscle cells were lysed in RIPA lysis buffer and incubated for 30 min on ice. The supernatant was collected after centrifugation. VSMCs in the media layer of mice were extracted as follows: mice arteries were opened longitudinally, and the endothelium and vascular adventitia were scraped off, then lysed with lysis buffer. Protein concentrations were tested by bicinchoninic acid assays (Beyotime, Shanghai, China). Proteins were separated by SDS-polyacrylamide gels and transferred onto PVDF membranes. The blots were blocked with 5% nonfat milk in 0.05% tween 20 in PBS and incubated with primary antibodies overnight at 4°C. Following incubation overnight, the blots were incubated with horseradish peroxidase-conjugated secondary antibodies and detected by an enhanced chemiluminescence detection kit.

### Molecular Docking Analysis

To investigate the probable binding conformation of Paeonol to the active site of AMPK, molecular docking studies were performed using the Surflex-Dock program (Jain, 1996) in SYBYL7.1 (Tripos, Inc., St. Louis, MO, United States). The crystal structures of human AMPK [PDB ID: 4CFE (Xiao et al., 2013), resolution = 3.02 Å] used for docking were obtained from the RSCB Protein Data Bank^1^. Water molecules and other heteroatoms were removed from the protein and hydrogen atoms were added subsequently. The 3D structure of Paeonol was prepared in MOL2 format and docked into the protein after energy was minimized. The default parameters of the Surflex-Dock program were used. Finally, the conformation with the highest-scored conformation was selected for studying the interactions between AMPK and Paeonol.

### Statistical Analysis

At least three independent replications were performed. Data are reported as the mean ± standard deviation (SD). Images were processed by use of Graphpad Prism 5 (GraphPad Software, La Jolla, CA, United States) and Adobe Photoshop (Adobe, San Jose, CA, United States). Student’s *t*-test was used for analysis between two groups with only one factor involved. A one-way ANOVA was used for analysis when more than two treatments were compared. Significant differences were established at *p* < 0.05.

## Results

### Paeonol Restricted Atherosclerosis Development in ApoE^-/-^ Mice

**FIGURE 1:**
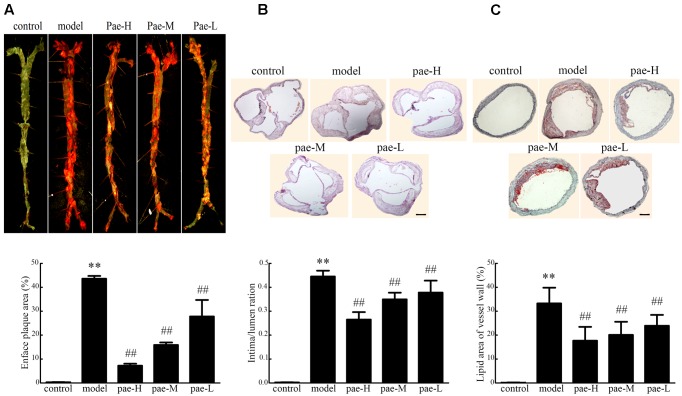
Effect of Paeonol on arterial atherosclerotic plaque development in apoE^-/-^ mice. **(A)** En face Oil-red O (ORO) staining of arteries and quantification. Bar = 3 mm, *n* = 5. **(B)** H&E staining and quantification of plaque areas. Bar = 500 μm. **(C)** ORO staining of atherosclerotic lesions. Bar = 500 μm. Data are the mean ± SD. ^∗∗^*P* < 0.01 vs. control group, ^#^*P* < 0.05, ^##^*P* < 0.01 vs. model group. Pae-H, 400 mg/kg body weight of Paeonol. Pae-M, 200 mg/kg body weight of Paeonol. Pae-L, 100 mg/kg body weight of Paeonol.

To evaluate the effect of Paeonol on atherogenesis, our group tested a range of indicators of atherosclerosis in apoE^-/-^ mice. In the Paeonol-treated group, plaque size was moderately lower compared to the model group and according to the en face ORO-staining (**Figure 1A**). Similar results were obtained from the H&E-stained frozen sections of the arterial sinus and ORO-stained frozen sections of the arterial roots (**Figures 1B,C**). Therefore, Paeonol treatment restricted atherosclerosis lesion development in apoE^-/-^ mice.

### Paeonol Inhibited Proliferation and Induced Autophagy in the Arterial Media Layer of ApoE^-/-^ Mice

**FIGURE 2:**
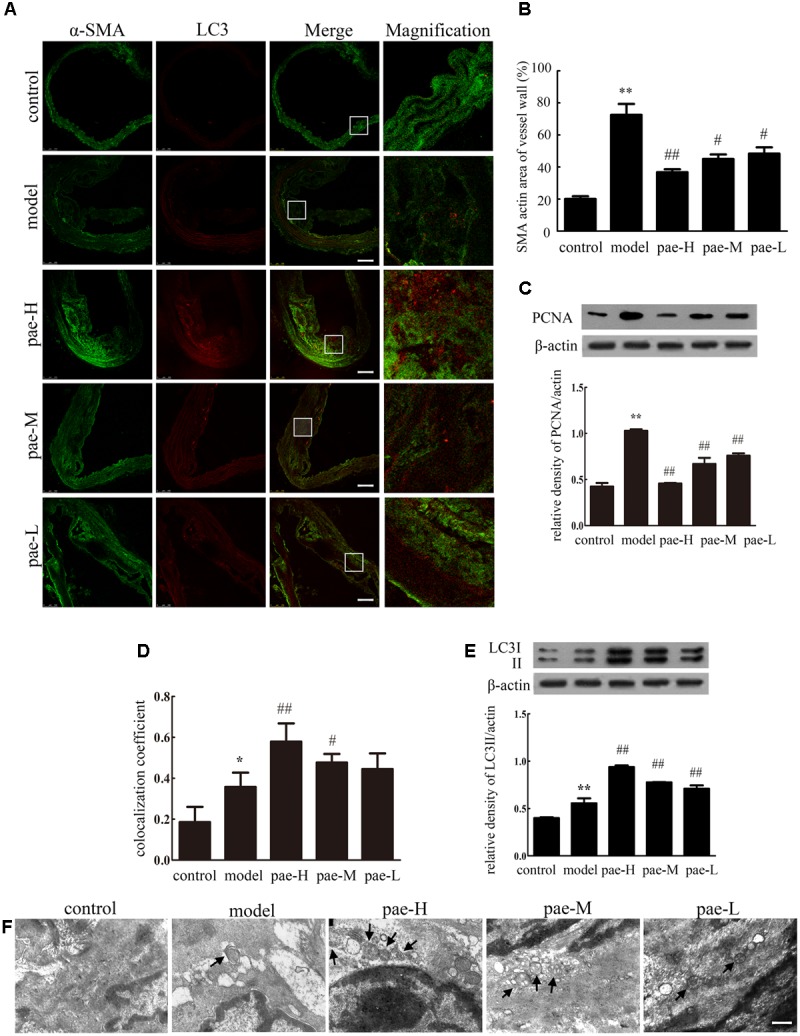
Paeonol induced vascular smooth muscle cell (VSMC) autophagy and inhibited cell proliferation in apoE^-/-^ mice. **(A)** The arteries were dissected and used for confocal immunofluorescent analysis. Representative confocal fluorescent images of the autophagy marker LC3II (red) with the smooth muscle marker α-SMA (green). Bar = 100 μm. **(B)** Quantification of smooth muscle area. **(C)** Protein expression of PCNA in the arterial media layer of mice. **(D)** Summarized colocalization coefficient of LC3II with α-SMA in the arterial media layer of mice. **(E)** Protein expression of LC3II in the arterial media layer of mice. Data are the mean ± SD, *n* = 3. ^∗∗^*P* < 0.01, ^∗^*P* < 0.05 vs. control group, ^#^*P* < 0.05, ^##^*P* < 0.01 vs. model group. **(F)** Electron microscope analysis of the arterial media layer of mice. Black arrows indicate autophagosomes. Bar = 200 nm. Pae-H, 400 mg/kg body weight of Paeonol. Pae-M, 200 mg/kg body weight of Paeonol. Pae-L, 100 mg/kg body weight of Paeonol.

Physiologically, VSMCs are the sole cell type of the media layer of the vascular wall. It has been well established that the role of VSMCs in atherosclerosis relates to their proliferative properties. VSMCs proliferate, grow and migrate into the intima to induce atherosclerotic plaque formation. Therefore, we examined the effect of Paeonol on VSMC proliferation using α-SMA staining in the media layer of apoE^-/-^ mice. As shown in **Figures 2A,B**, Paeonol decreased the level of α-SMA in the media layer of apoE^-/-^ mice. Western blotting on artery tissue demonstrated that the expression of PCNA was highly decreased in the Paeonol-treated mice compared to that of the model groups (**Figure 2C** and **Supplementary Figure S1a**). Together, these results confirmed that Paeonol inhibited VSMC proliferation and prevented vascular fibrosis *in vivo*. This might be one reason why Paeonol can restrict atherosclerosis development in apoE^-/-^ mice.

In the early stage of atherogenesis, enhanced autophagy in VSMCs is favorable to decreasing cell proliferation and preventing fibrosis. We also examined the effect of Paeonol on autophagy by staining LC3II spots in the plaque media layer of apoE^-/-^ mice. As shown in **Figures 2A,D**, representative confocal microscopic images and the summarized colocalization coefficients demonstrate that LC3II increased much more in arterial media of Paeonol-treated mice compared with model mice, in which it colocalized mainly with the smooth muscle marker α-SMA. Immunofluorescence assays revealed that Paeonol increased the protein level of LC3II in the plaque media layer of apoE^-/-^ mice, which confirmed that Paeonol induced VSMC autophagy *in vivo*. Western blotting on tissue extracts of the media layer of arteries demonstrated that the expression of LC3II was highly increased in the Paeonol-treated mice compared to that of model groups (**Figure 2E** and **Supplementary Figure S1b**). This result was further confirmed by TEM, which also showed an increased presence of autophagosomes in the media layer of apoE^-/-^ mice from pae-L, pae-M, and pae-H groups (**Figure 2F**).

Compared with the model group, the media layer of apoE^-/-^ mice showed inhibited VSMC proliferation and increased autophagy with Paeonol treatment; therefore, Paeonol induced VSMC autophagy and protected against cell proliferation in atherosclerosis.

### Paeonol Inhibited ox-LDL-Induced Vascular Smooth Muscle Cell Proliferation

**FIGURE 3:**
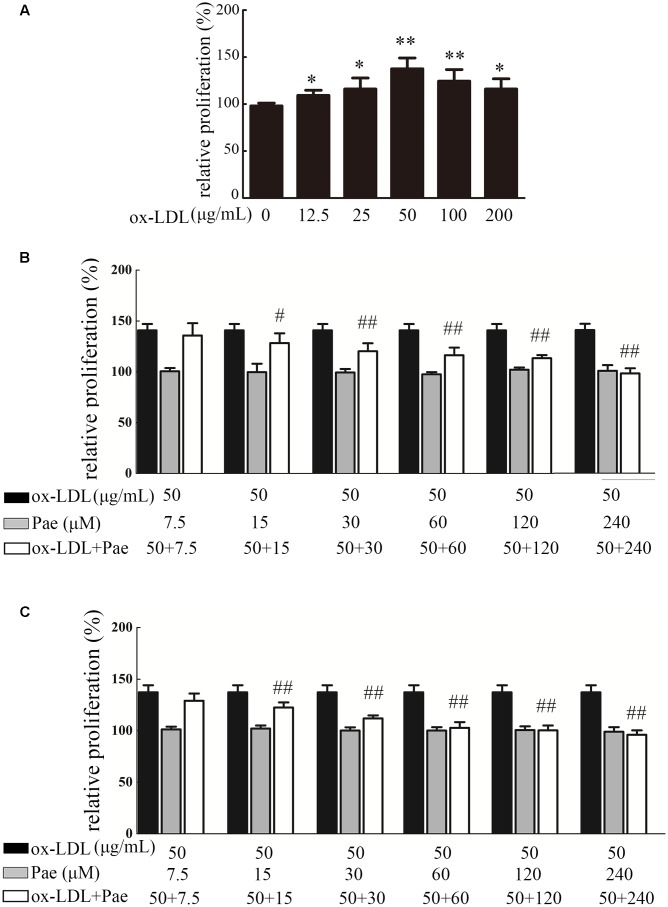
Paeonol treatment prevents VSMC proliferation and DNA synthesis by ox-LDL stimulation. **(A)** Effect of different concentrations of ox-LDL on VSMCs proliferation, as assessed by a CCK8 assay. **(B)** VSMCs were treated with the indicated concentration of Paeonol (7.5–240 μM) for 24 h in the absence or presence of ox-LDL (50 μg/mL). Cell viability was examined with CCK8 assay. **(C)** Cell proliferation was detected by BrdU assay. Data are the mean ± SD, *n* = 3. ^∗^*p* < 0.05, ^∗∗^*p* < 0.01 vs. control group. ^#^*p* < 0.05, ^##^*p* < 0.01 vs. ox-LDL group.

**Figure 3A** shows the effect of the ox-LDL concentration (12.5–200 μg/mL) on cellular proliferation by CCK-8 assay. As shown in **Figure 3A**, treatment with ox-LDL (12.5–200 μg/mL) for 24 h resulted in increased cell proliferation, which reached its highest value at 50 μg/mL ox-LDL. Ox-LDL concentrations beyond 200 μg/mL showed obvious cytotoxic effects. In this study, treatment with 50 μg/mL ox-LDL for 24 h was chosen for use in subsequent experiments. We first investigated the effect of Paeonol on cell proliferation using a CCK8 assay. Paeonol markedly inhibited VSMC proliferation in a dose-dependent manner (**Figure 3B**). BrdU incorporation assays were used to further investigate the effects of Paeonol on DNA synthesis. Paeonol significantly inhibited the BrdU incorporation induced by ox-LDL stimulation in a dose-dependent manner (**Figure 3C**). Moreover, Paeonol treatment in the absence of ox-LDL did not decrease the viability of VSMCs or their incorporation of BrdU, indicating that Paeonol treatment at these concentrations has no cytotoxic effect on cells; thus, it is an inhibitory effect of Paeonol n target DNA synthesis rather than cytotoxicity that causes the loss of cellular DNA (**Figures 3B,C**).

### Paeonol Arrested VSMCs in the G1/G0 Phase of the Cell Cycle

**FIGURE 4:**
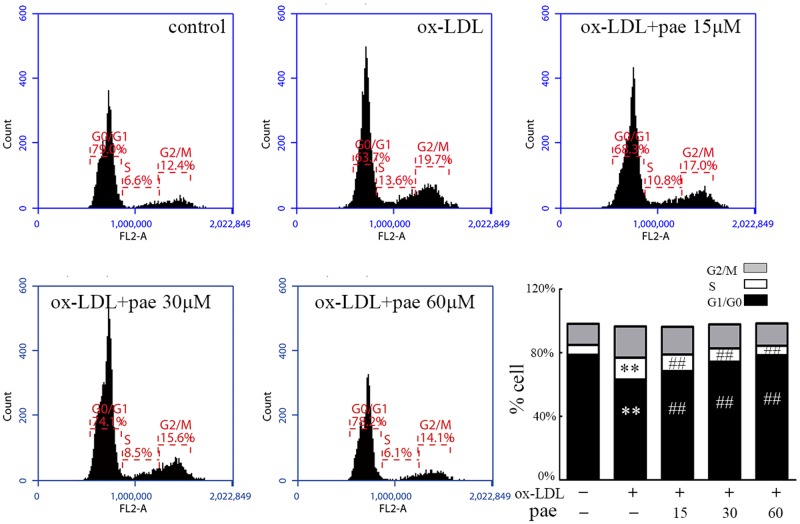
Paeonol treatment prevents cell cycle progression in VSMCs. VSMCs were treated with different concentrations of Paeonol in the absence or presence of ox-LDL (50 μg/mL) for 24 h before cell nuclei were stained with PI and analyzed by flow cytometry. A compiled evaluation of three independent experiments is shown. Data are the mean ± SD, *n* = 3. ^∗∗^*p* < 0.01 vs. control group. ^##^*p* < 0.01 vs. ox-LDL group.

Cell proliferation is controlled by progression through the cell cycle. We analyzed the effects of Paeonol treatment on cell cycle stage distribution of VSMCs. As shown by flow cytometry (**Figure 4**), ox-LDL stimulation significantly increased the percentage of cells in the S phase and decreased the percentage of those in the G0/G1 phases, whereas Paeonol significantly decreased the number of S-phase cells and increased the fraction of G0/G1 phases in the VSMCs. Notably, the Paeonol-mediated arrest in the S phase was also consistent with the observed reduction in BrdU incorporation occurring in the S phase of the cell cycle.

### Paeonol Induced VSMCs Autophagy

**FIGURE 5:**
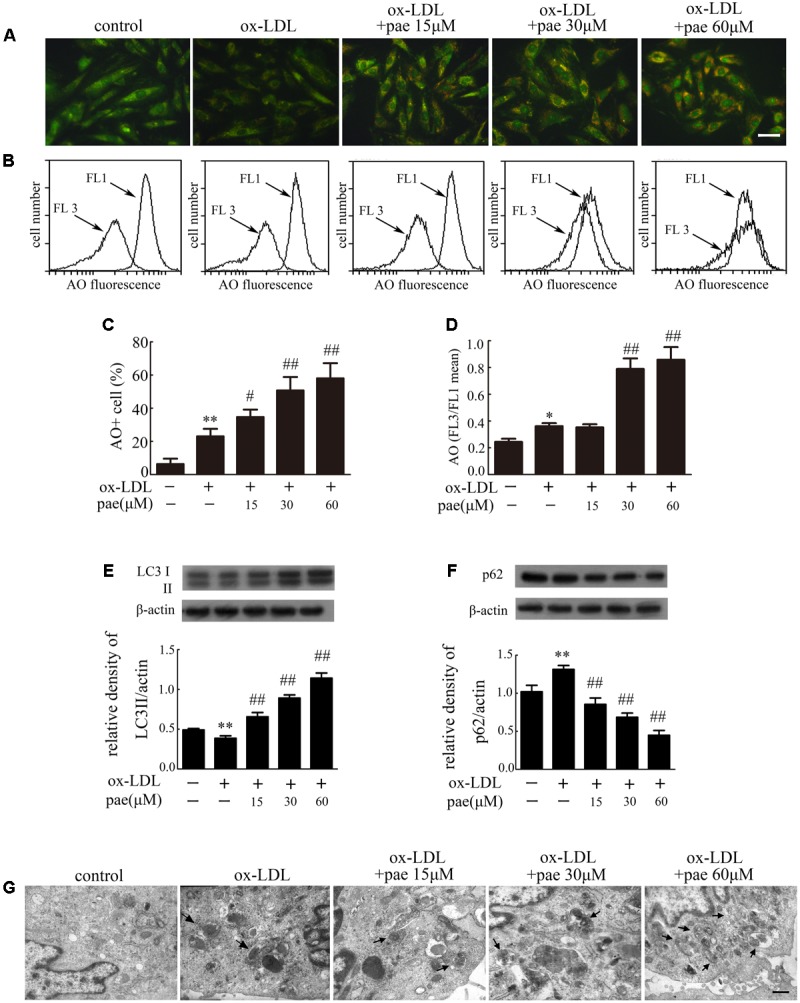
Paeonol induced autophagy in VMSCs. Cells were treated with different concentrations of Paeonol in the absence or presence of ox-LDL (50 μg/mL) for 24 h. The AO-staining in cells was demonstrated by fluorescence microscopy, Bar = 50 μm **(A)** and flow cytometry **(B)**. **(C)** Autophagic values were calculated as the percentage of AO^+^ cells relative to the total number of cells. **(D)** Intracellular acidification was calculated by red-to-green (FL3/FL1) mean fluorescence intensity. **(E)** LC3 processing and **(F)**, p62 levels in VSMCs were estimated by western blot assay. Data are the mean ± SD, *n* = 3. ^∗∗^*p* < 0.01 vs. control group. ^#^*p* < 0.05, ^##^*p* < 0.01 vs. ox-LDL group. **(G)** Representative TEM images of VSMCs. Arrows indicated the autophagosome. Bar = 500 nm.

The molecular events responsible for activating autophagic mechanisms were further studied after ox-LDL treatment together with or without Paeonol. Both fluorescence microscopy and flow cytometry analyses demonstrated an increase in AO red fluorescence in Paeonol treated cells (**Figures 5A–D**). The addition of Paeonol to the ox-LDL treatment induced a dose-dependent synergistic effect on autophagic levels, as evidenced by an increase in the formation of autophagic vacuoles. The autophagic levels in VSMCs were also evaluated by detection of LC3 processing and LC3II accumulation by immunoblot analysis. Accordingly, Paeonol in a dose-dependent manner increased the ratio of LC3II/actin (**Figure 5E** and **Supplementary Figure S2a**), indicating that Paeonol induced autophagy in VSMCs. Treatment with Paeonol observably decreased the level of p62 (**Figure 5F** and **Supplementary Figure S2b**), an index of autophagic degradation (Ichimura et al., 2008), which actually represented an increase in autophagy mediated proteolysis. Finally, the induction of autophagy was confirmed by TEM analysis. TEM images in the control and ox-LDL group displayed normal cytoplasm, characterized by mitochondria, an irregular nucleus, and free ribosomes, as well as autophagosomes and lysosomes. In contrast, the Paeonol group showed extensive cytoplasmic vacuolization with many autophagosomes in the cytoplasm at different stages (**Figure 5G**).

### Paeonol Prevented ox-LDL-Induced VSMC Proliferation via Up-regulation of Autophagy

To investigate if Paeonol could prevent VSMCs proliferation through autophagy induction, we tested the change in autophagy and cell proliferation by inhibition of autophagy. VSMCs were pretreated with CQ for 1h and treated with ox-LDL (50 μg/mL) in the absence or presence of Paeonol (30 μM) for 24 h. The levels of LC3II accumulation and p62 degradation were assessed by immunoblot in the absence or presence of CQ.

**FIGURE 6:**
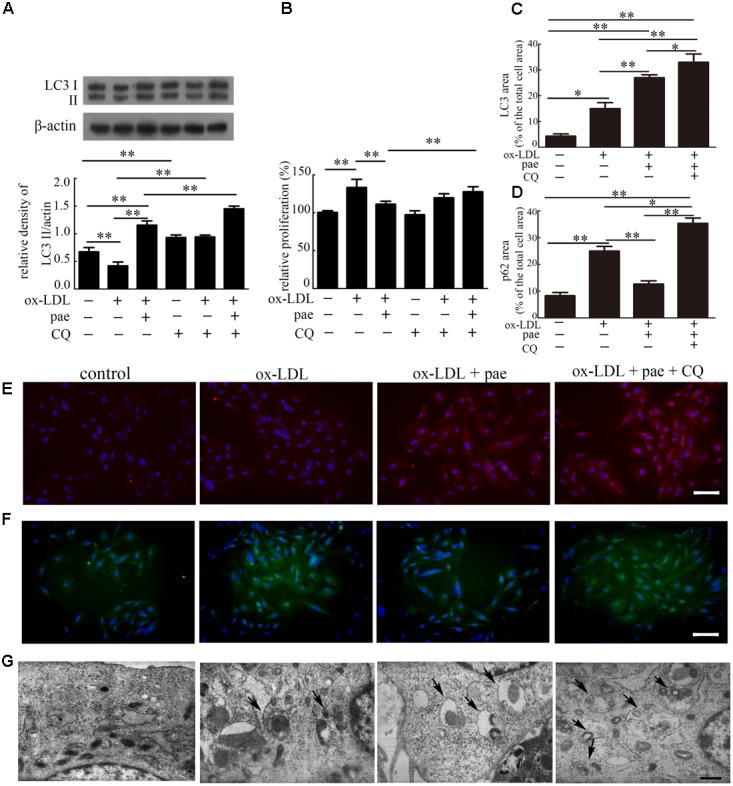
Paeonol inhibited ox-LDL induced cell proliferation through the induction of autophagy in VSMCs. **(A)** Representative blots of LC3 processing in ox-LDL-induced VSMCs treated with Paeonol in the absence or presence of CQ. **(B)** VSMCs proliferation was analyzed by CCK8 assay. **(C)** The percentage of LC3-positive dots in the total cell area. **(D)** The percentage of p62-positive dots in the total cell area. Data are the mean ± SD, *n* = 3. ^∗^*p* < 0.05, ^∗∗^*p* < 0.01. **(E)** Representative image of immunofluorescence stained by LC3 (red) and DAPI. **(F)** Representative image of immunofluorescence stained by p62 (green) and DAPI. Bar = 50 μm. **(G)** TEM images of VSMCs. Arrows indicate the autophagosomes. Bar = 500 nm.

Compared with the control group, CQ alone caused an obvious increase in LC3II expression, which reflected autophagosome accumulation because of autophagic flux inhibition. The addition of CQ to the ox-LDL and Paeonol-combined treated group resulted in a higher level of LC3II than that in the ox-LDL and Paeonol combined treated group. Compared with the control or ox-LDL groups, Paeonol obviously increased LC3II accumulation (**Figure 6A** and **Supplementary Figure S3**). In addition, after Paeonol-induced autophagy was inhibited by CQ, the anti-proliferation effects of Paeonol were lost, as assessed using a CCK8 assay (**Figure 6B**). Compared with the ox-LDL group, pretreatment of CQ in the ox-LDL alone group showed no influence on cell proliferation. These results showed that the anti-proliferation effects of Paeonol contributed to autophagy in VSMCs.

To further confirm Paeonol-induced autophagy in VSMCs, immunofluorescence was utilized to reveal the intracellular localization of LC3 and p62. VSMCs in the control and ox-LDL groups showed little staining of LC3 (red). In contrast, VSMCs treated with Paeonol or Paeonol with CQ displayed a positive staining distribution of LC3 (**Figures 6C,E**). For p62, VSMCs treated with Paeonol showed little staining of p62 (**Figures 6D,F**). Ultrastructural changes in VSMCs were also detected with TEM (**Figure 6G**). VSMCs treated with Paeonol or Paeonol with CQ displayed autophagosomes dispersed in cytoplasm.

### Paeonol Activated Autophagy via AMPK/mTOR Pathway in VSMCs

To explore the potential molecular mechanisms by which Paeonol induced autophagy, we assessed autophagy up-regulation via the AMPK/mTOR signaling pathway.

**FIGURE 7:**
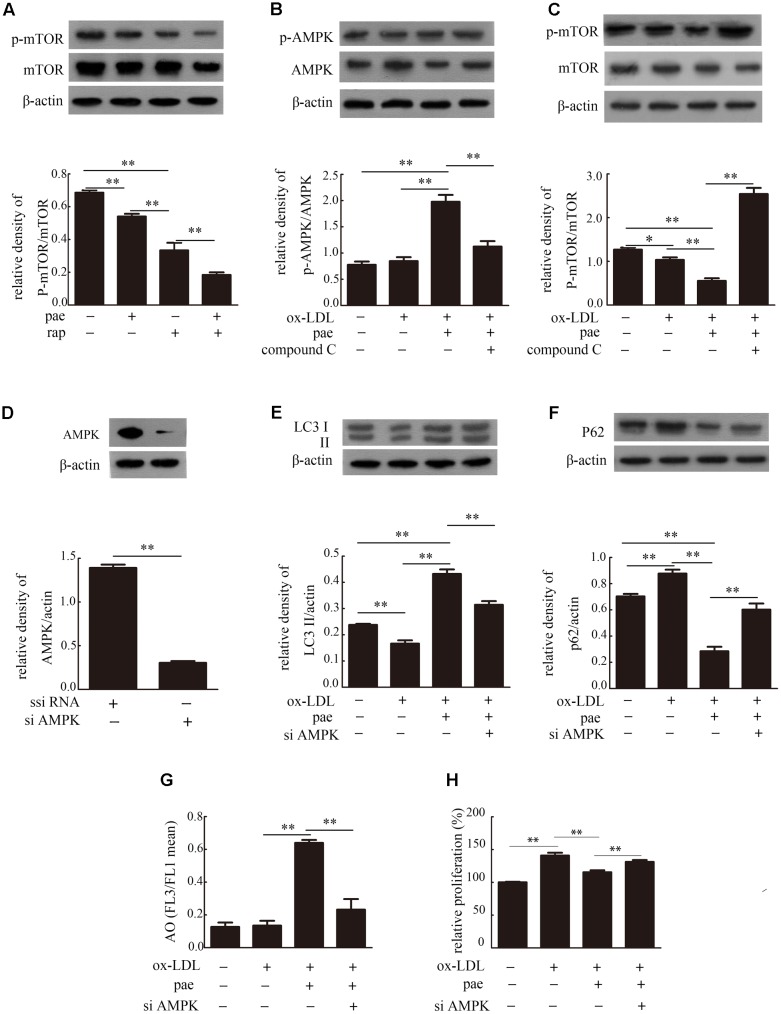
Paeonol induced autophagy in VSMCs was via the AMPK/mTOR signaling pathway. **(A)** Representative blots of p-mTOR/mTOR in VSMCs treated with Paeonol in the absence or presence of rap. Representative blots of p-AMPK/AMPK **(B)** and p-mTOR/mTOR **(C)** in ox-LDL-induced VSMCs treated with Paeonol in the absence or presence of compound C. **(D)** Immunoblot verification of AMPK knockdown. Representative blots of LC3II **(E)** and p62 **(F)** in VSMCs or si-AMPK transfected cells treated with ox-LDL and Paeonol. **(G)** Intracellular acidification was calculated by red-to-green (FL3/FL1) mean fluorescence intensity. **(H)** VSMC proliferation was analyzed by a CCK8 assay. Data are the mean ± SD, *n* = 3. ^∗^*p* < 0.05, ^∗∗^*p* < 0.01.

VSMCs were incubated with rapamycin (rap, 100 nM, a mTOR inhibitor) in the absence or presence of 30 μM Paeonol. Rapamycin decreased the ratio of p-mTOR/mTOR which was reduced more with the incubated with Paeonol in the presence of the rapamycin (**Figure 7A** and **Supplementary Figure S4a**). We also detected the levels of AMPK in autophagy regulation. AMPK phosphorylation at position Thr172 was determined by immunoblot with an anti-phosphor-AMPK antibody. AMPK phosphorylation was significantly increased after Paeonol treatment in VSMCs (**Figure 7B** and **Supplementary Figure S4b**). As shown in **Figure 7C** (**Supplementary Figure S4b**), we found that the ratio of p-mTOR/mTOR was decreased by Paeonol treatment and increased by addition of compound C. To confirm that AMPK activity plays a key role in autophagy regulation of Paeonol, we used si-AMPK transfection and detected efficient transfection in VSMCs (**Figure 7D** and **Supplementary Figure S4c**). We found that Paeonol-induced LC3 conversion and p62 degradation were reversed in AMPK-deficient cells (**Figures 7E,F** and **Supplementary Figure S4d**). In addition, treatment with Paeonol obviously induced intracellular acidification and inhibited proliferation in VSMCs, but not in AMPK-deficient cells (**Figures 7G,H**). Therefore, these results suggested that the AMPK/mTOR signaling pathway is involved in Paeonol-induced autophagy.

**FIGURE 8:**
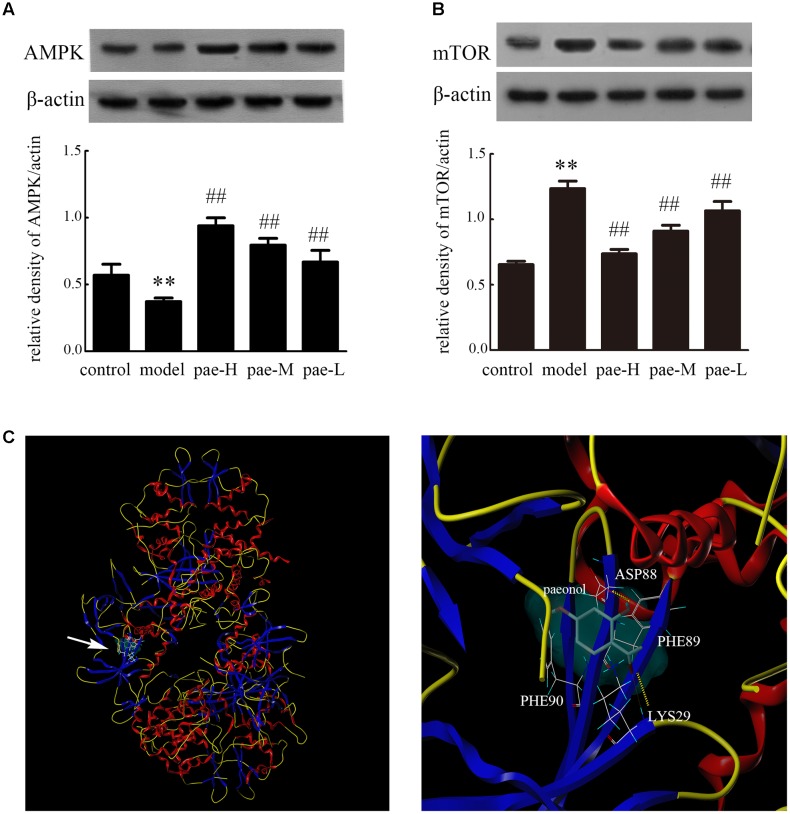
Effect of Paeonol on APMK/mTOR expression in the arterial media layer of apoE^-/-^ mice. Protein expression of AMPK **(A)** and mTOR **(B)** in the arterial media layer of mice. Data are the mean ± SD, *n* = 3. ^∗∗^*p* < 0.01 vs. control group. ^##^*p* < 0.01 vs. model group. **(C)** Molecular docking of Paeonol targeting AMPK.

Furthermore, western blotting of tissue extracts from the media layer of arteries in apoE^-/-^ mice demonstrated that Paeonol obviously induced expression of AMPK and mTOR down-regulation compared to that of the model group (**Figures 8A,B** and **Supplementary Figure S5**). The results confirmed that Paeonol can significantly activate the AMPK/mTOR signaling pathway. To further explore how Paeonol activates AMPK, molecular docking studies were performed. The best possible binding mode between Paeonol and 4CFE is presented in **Figure 8C**. As shown in **Figure 8C**, some key amino acids (ASP88, PHE89, PHE90, and LYS29) in the binding pocket interact with Paeonol by hydrogen bonds and p-p conjugation. The O atom at the carbonyl group of Paeonol forms an H-bond (–O…HN–) with LYS29, and the –OH group of Paeonol forms an H-bond (–OH…O–) with ASP88. In addition, the aromatic ring side chain of PHE89 and PHE90 participate in hydrophobic interactions with Paeonol.

## Discussion

The important findings of this research were the anti-atherosclerotic effects of Paeonol against VSMC proliferation through up-regulating autophagy via activation of the AMPK/mTOR signaling pathway *in vivo* and *in vitro*. We observed that Paeonol has anti-atherosclerotic effects, as demonstrated by the evident improvement of the reduced plaque area and the intima/lumen ration, and decreased the number of VSMCs in apoE^-/-^ mice arteries. Next, we found that Paeonol induced autophagy, as demonstrated by an increased autophagosome and intracellular acidification, and enhanced LC3 conversion and p62 degradation in VSMCs and apoE^-/-^ mice arteries. Most importantly, these beneficial effects of Paeonol were likely achieved by up-regulating autophagy via the autophagy-lysosome pathway, as the anti-proliferative effects of Paeonol could be attenuated by co-treatment with CQ, an autophagy inhibitor. In addition, the anti-proliferative effects of Paeonol were obviously abolished in compound C treated or AMPK-deficient VSMCs, which further indicated that the activation of the AMPK/mTOR signaling pathway was involved in Paeonol-induced autophagy in VSMCs.

Paeonol, a natural compound from moutan cortex, has various medicinal properties that have been used extensively in traditional Chinese medicine. In previous studies, our group demonstrated that Paeonol can attenuate atherosclerosis plaque development in quails and rabbits (Dai et al., 1999; Li et al., 2009), and that Paeonol can prevent endothelial cell injury and vascular smooth muscle cell proliferation *in vitro* (Liu et al., 2014; Wu et al., 2015). ApoE^-/-^ mice have been shown to develop severe hypercholesterolemia and atherosclerosis lesions that are more characteristic in appearance and distribution to those observed in humans (Nakashima et al., 1993). In this study, the athero-protective effects of Paeonol were examined in apoE^-/-^ mice. *In vivo* experiments confirmed that Paeonol attenuated atherosclerosis plaque development and restricted atherosclerosis development.

The media layers of arteries, which are comprised of VSMCs, are heavily involved in the development of vascular diseases. In atherosclerosis, LDL and its oxidized forms accumulate in the subintimal space, which recruits monocytes and provokes the proliferation and migration of VSMCs (Salabei and Hill, 2015). Since VSMCs form a significant constituent of the atherosclerotic lesion, we focused on this cell type to study the effects of Paeonol on the development of atherosclerosis *in vivo*. We found that Paeonol decreased the number of VSMCs and increased the protein levels of LC3 and the presence of autophagosomes in the arterial media layer of apoE^-/-^ mice, which implied that Paeonol may induce VSMCs autophagy *in vivo*.

Previous studies demonstrated that autophagy plays a key role in the pathogenesis of atherosclerosis. Defective autophagy in VSMCs disturbed cell homeostasis and induced cell proliferation, which finally led to and even accelerated atherogenesis (Grootaert et al., 2015). Induction of autophagy inhibited thrombin-induced VSMCs proliferation through the autophagy-lysosome pathway (Dong et al., 2012). Several studies reported that autophagy stimulation combined with radiotherapy or chemotherapy has been used as a promising treatment option in cancer therapy because of its anti-proliferation effects in cancer cells (Pimkina and Murphy, 2009). Anti-proliferative treatment options depending on autophagy regulation in VSMCs may be a promising avenue for atherosclerosis therapy.

The possible role of Paeonol in suppressing VSMCs proliferation related to autophagy up-regulation has yet to be elucidated. In our research, we first detected that Paeonol inhibited VSMCs proliferation via an autophagy-dependent mechanism. These indexes, such as autophagosome amount, cytoplasmic acidification, LC3 conversion and p62 degradation, were utilized to evaluate autophagic levels at various stages in VSMCs. We used ox-LDL, an established risk factor (Yamada et al., 1998; Luo et al., 2010), to activate VSMC proliferation *in vitro*. In accordance with previous studies, 50μg/mL ox-LDL obviously caused proliferation of VSMCs (Ding et al., 2012). We found an antiproliferative effect of Paeonol *in vitro*, which was in accordance with other studies on the Paeonol-induced antiproliferative effects in VSMCs and in non-VSMCs (Chen et al., 2014; Xu et al., 2017). Our findings also indicated that autophagy was activated by 50μg/mL ox-LDL in VSMCs, which was demonstrated by the increased ratio of LC3II/actin and supported by results of former studies (Ding et al., 2013). In contrast, ox-LDL treatment led to increased p62 expression, which indicated that ox-LDL could block autophagic flux. Several documents indicated a significant inverse correlation between p62 expression and autophagic flux, which means that p62 is one of the indexes required for complete autophagy (Kabeya et al., 2000). In this study, Paeonol further up-regulated LC3II conversion and reduced p62 expression compared to the ox-LDL group. Previous studies indicated that down-regulation of autophagy activated nuclear factor-κB signaling (Ai et al., 2012), while in our recent research we found that Paeonol blocked the activation of the PI3K/Akt/NF-κB signaling pathway (Yuan et al., 2016). These results indicated that Paeonol may up-regulate cell autophagy, which was subsequently confirmed by the data in this study. Several studies indicated that induced autophagy caused a reversible reduction of cell proliferation in VSMCs or in other cell lines (Stamatiou et al., 2009; Salabei et al., 2012; Tang et al., 2014). After Paeonol-induced autophagy was inhibited by CQ, the anti-proliferation effects of Paeonol were lost. These results confirmed that Paeonol inhibited VSMC proliferation through up-regulation of autophagy via the autophagy-lysosome pathway.

To clarify the autophagy-inducing mechanism of Paeonol, we detected the effectiveness of AMPK/mTOR signaling activation in the up-regulation of autophagy. AMPK/mTOR is an important participant in the process of autophagy. AMPK was involved in endothelial protective effects of Paeonol, which halved the risk of cardiovascular disease (Choy et al., 2016). Paeonol possessed an anti-proliferation effect on human prostate cancer cells and significantly inhibited phosphorylation status of Akt and mTOR (Xu et al., 2017). These findings support our results that Paeonol increased the ratio of p-AMPK/AMPK and decreased the ratio of p-mTOR/mTOR in VSMCs. In our study, the phosphorylated status of mTOR was enhanced by the addition of compound C. More importantly, up-regulation of autophagy by Paeonol was abolished in si-AMPK VSMCs, which was demonstrated by a lower LC3 conversion and p62 degradation and a reduced cytoplasmic acidification level. This appearance implied that AMPK is upstream of mTOR signaling, and the AMPK/mTOR pathway was involved in the up-regulation autophagy by Paeonol in VSMCs. Molecular docking studies were performed and indicated that Paeonol might directly bind to AMPK.

## Conclusion

Paeonol could suppress atherosclerosis by inhibiting VSMCs proliferation via up-regulating autophagy through the AMPK/mTOR signaling pathway *in vivo* and *in vitro.* Our study offers novel insights into the anti-proliferation effect of Paeonol, which will provide references for clinic use of Paeonol for therapy of atherosclerosis. However, it will be a challenge to determine how to induce moderate autophagy in the human body, which will require more research.

## Author Contributions

HW performed the whole experiments and wrote the paper. AS and WH supported the Wu’s experiments, contributed to finishing bioassays and analyzing the data of biological activity. MD conceived and designed the experiments. All authors have read and approved the final version of the article.

## Conflict of Interest Statement

The authors declare that the research was conducted in the absence of any commercial or financial relationships that could be construed as a potential conflict of interest.

## Footnotes

1 www.rcsb.com

## Supplementary Material

FIGURE S1 The original blots images of **Figure 2**. **(a)** Original blots images of **Figure 2C**. **(b)** Original blots images of **Figure 2E**.

FIGURE S2 The original blots images of **Figure 5**. **(a)** Original blots images of **Figure 5E**. **(b)** Original blots images of **Figure 5F**.

FIGURE S3 The original blots images of **Figure 6A**.

FIGURE S4 The original blots images of **Figure 7**. **(a)** Original blots images of **Figure 7A**. **(b)** Original blots images of **Figures 7B,C**. **(c)** Original blots images of **Figure 7D**. **(d)** Original blots images of **Figures 7E,F**.

FIGURE S5 The original blots images of **Figures 8A,B**.
