# Supplementary figures and images for: The Anti-atherosclerotic Effect of Paeonol against Vascular Smooth Muscle Cell Proliferation by Up-regulation of Autophagy via the AMPK/mTOR Signaling Pathway
Source: Front Pharmacol. 2018 Jan 4;8:948. doi: 10.3389/fphar.2017.00948 (PMC5758604; doi:10.3389/fphar.2017.00948)

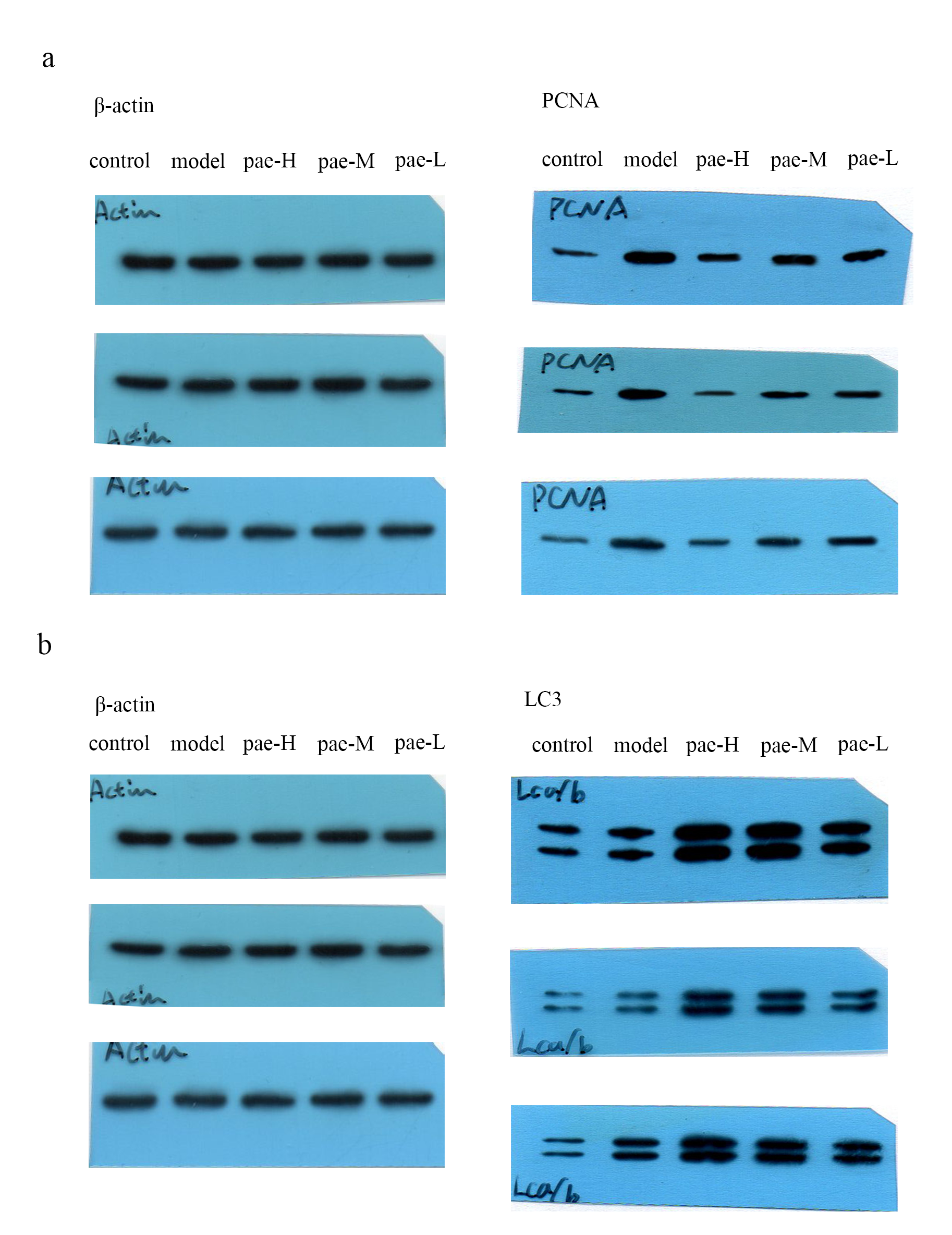

Supplement: FIGURE S1 — The original blots images of Figure 2. (a) Original blots images of Figure 2C. (b) Original blots images of Figure 2E. [file Image_1.tif]

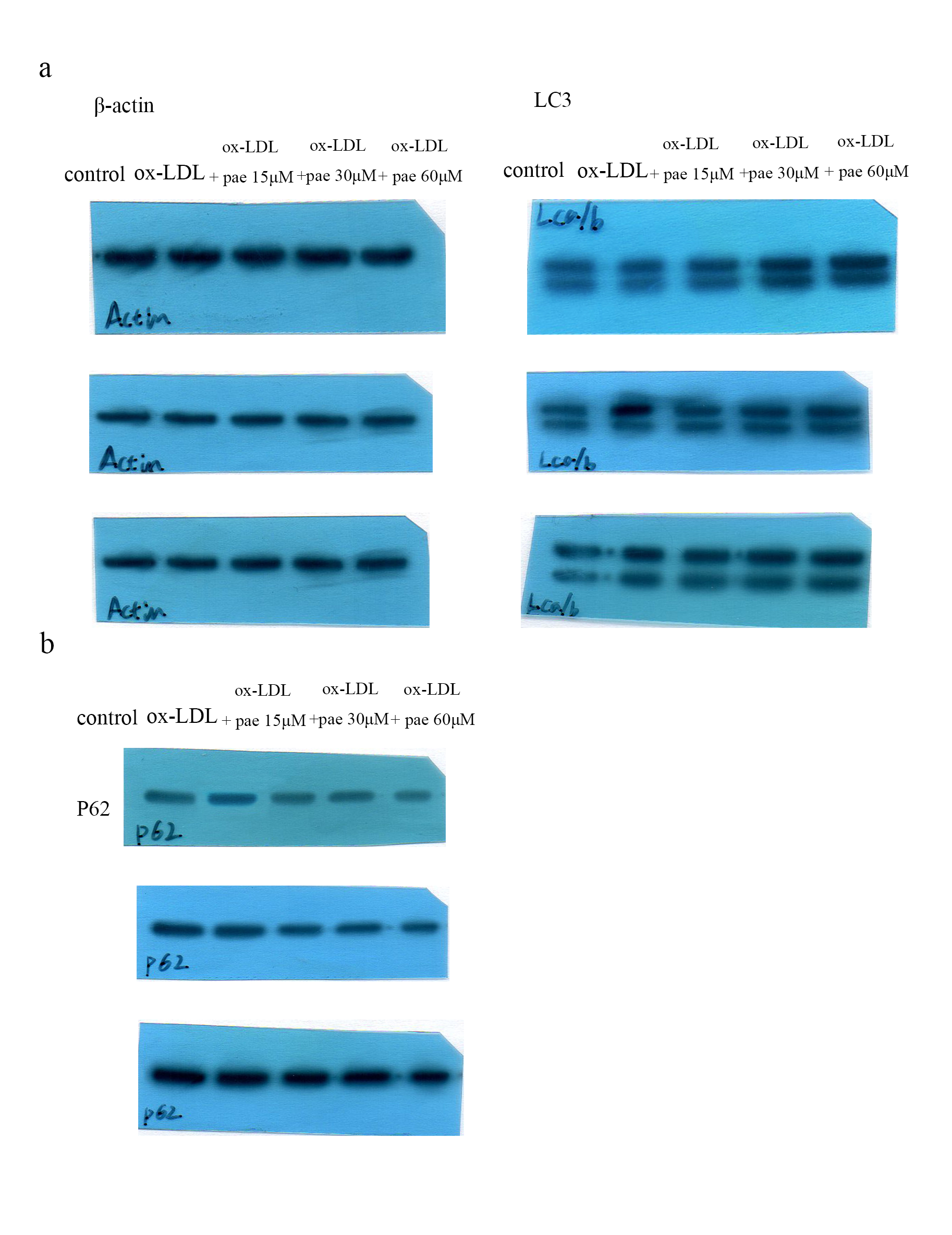

Supplement: FIGURE S2 — The original blots images of Figure 5. (a) Original blots images of Figure 5E. (b) Original blots images of Figure 5F. [file Image_2.tif]

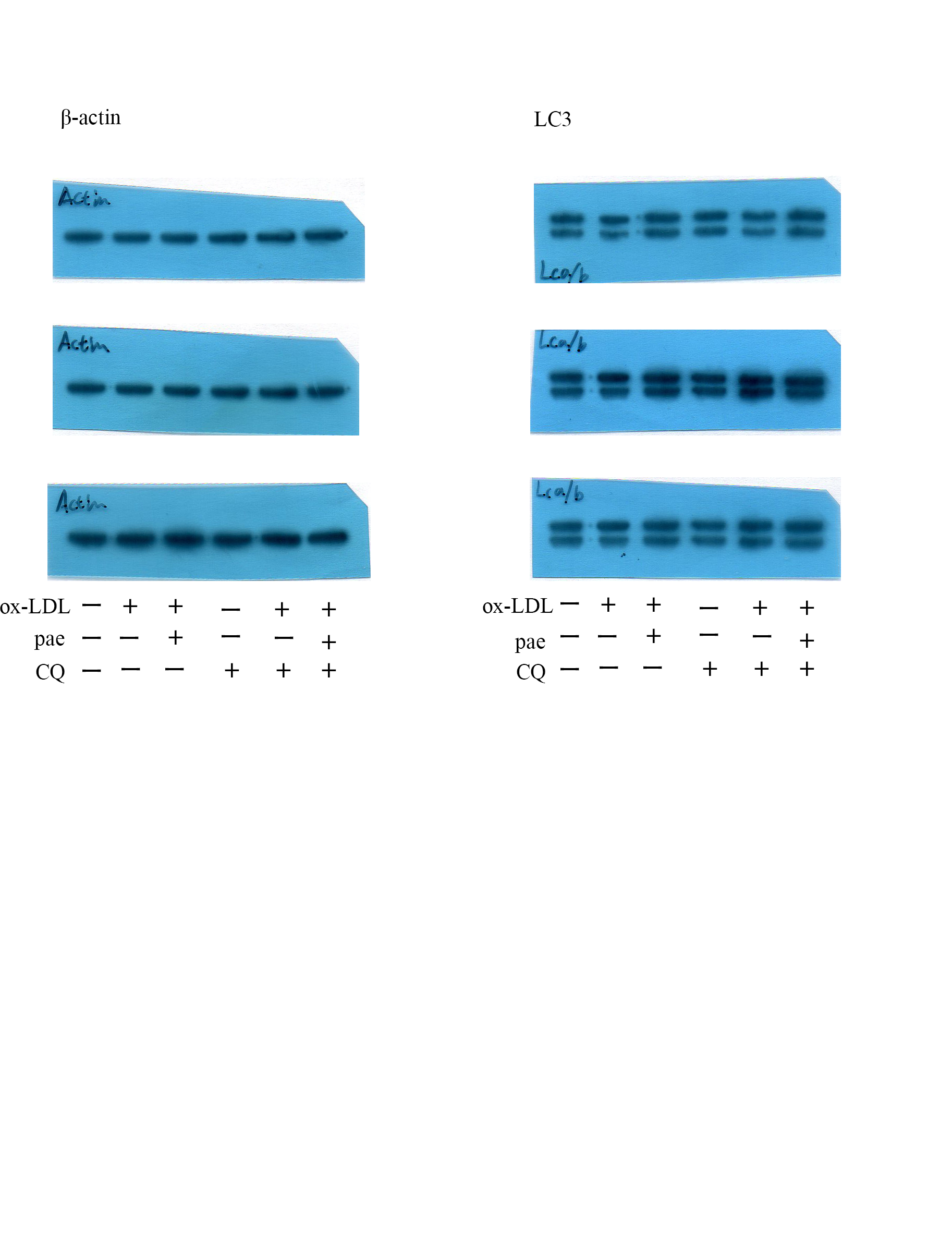

Supplement: FIGURE S3 — The original blots images of Figure 6A. [file Image_3.tif]

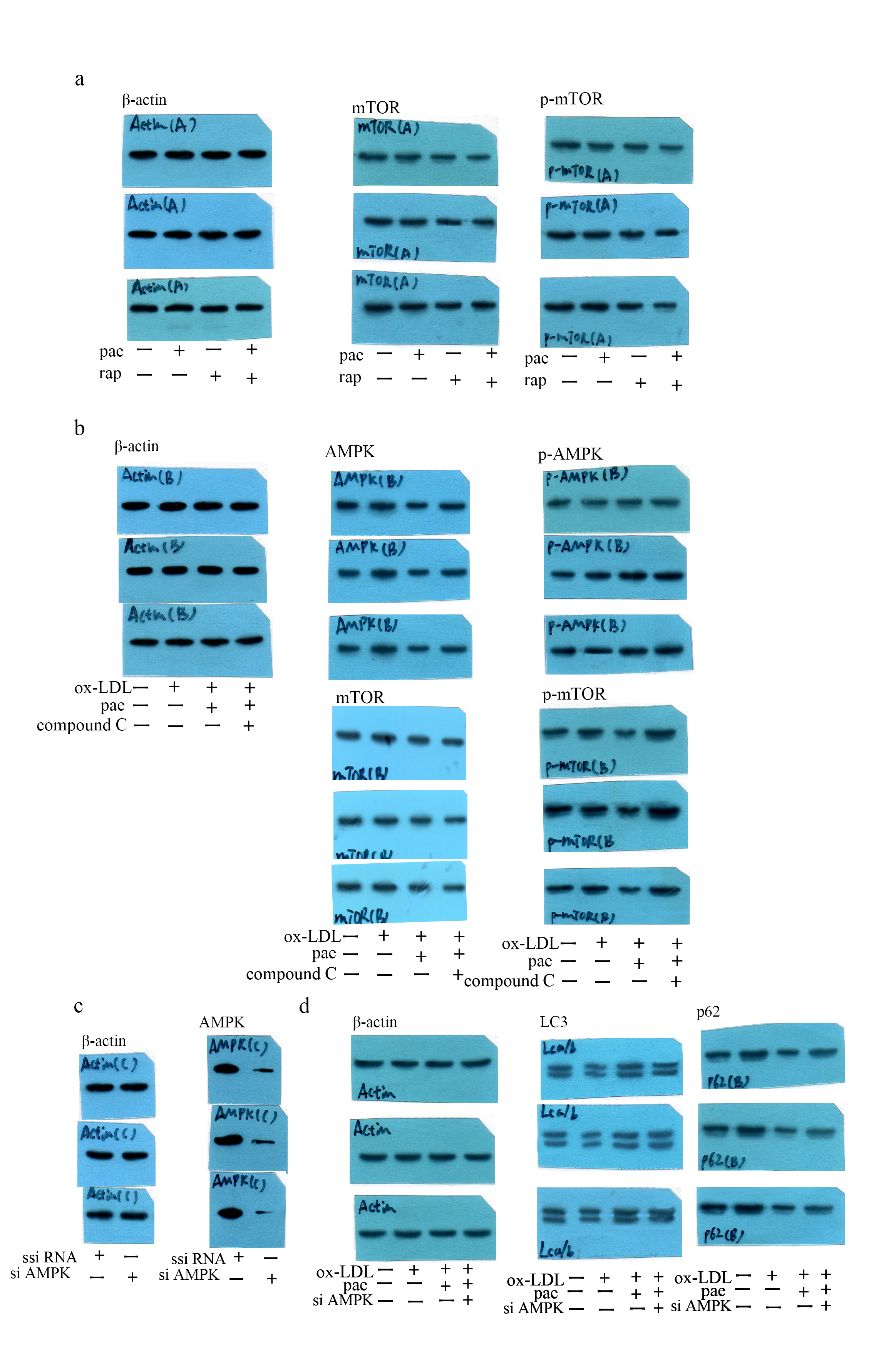

Supplement: FIGURE S4 — The original blots images of Figure 7. (a) Original blots images of Figure 7A. (b) Original blots images of Figures 7B,C. (c) Original blots images of Figure 7D. (d) Original blots images of Figures 7E,F. [file Image_4.tif]

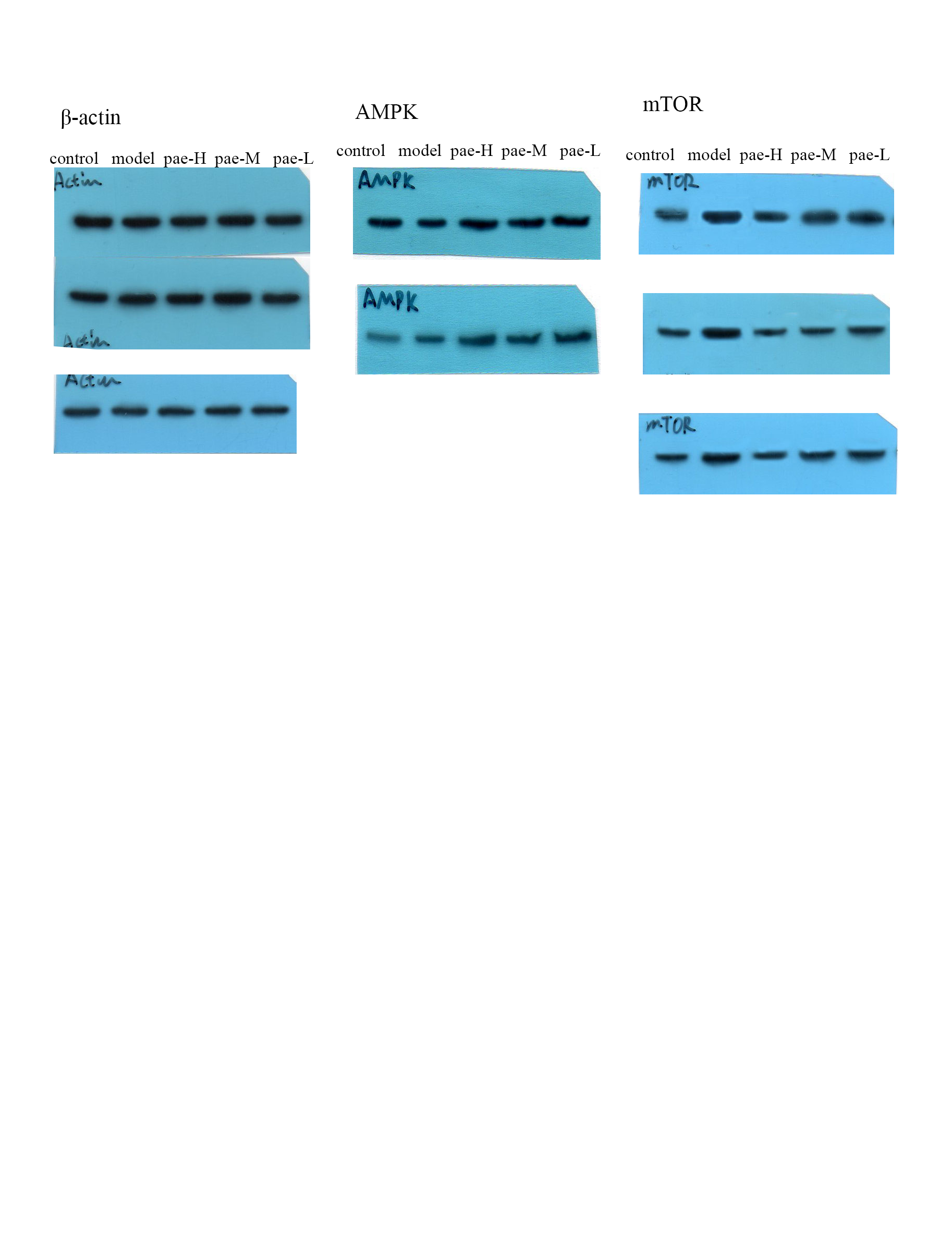

Supplement: FIGURE S5 — The original blots images of Figures 8A,B. [file Image_5.tif]
